# Exploring the Genetic Regulation of Asexual Sporulation in *Zymoseptoria tritici*

**DOI:** 10.3389/fmicb.2018.01859

**Published:** 2018-08-14

**Authors:** Anna M. M. Tiley, Gary D. Foster, Andy M. Bailey

**Affiliations:** Molecular Plant Pathology and Fungal Biology Group, School of Biological Sciences, University of Bristol, Bristol, United Kingdom

**Keywords:** *Zymoseptoria tritici*, *Aspergillus nidulans*, ascomycete, septoria tritici blotch, asexual sporulation, pycnidia, pycnidiospores

## Abstract

*Zymoseptoria tritici* is the causal agent of septoria tritici blotch, a devastating fungal disease of wheat which can cause up to 40% yield loss. One of the ways in which *Z. tritici* spreads in the field is via rain splash-dispersed asexual pycnidiospores, however there is currently limited understanding of the genetic mechanisms governing the development of these propagules. In order to explore whether the existing models for conidiation in ascomycete fungi apply to *Z. tritici*, homologs to the well-characterized *Aspergillus nidulans* genes *abacus* (*abaA*)*, bristle* (*brlA*)*, fluffy B* (*flbB*), *fluffy C* (*flbC*), and *stunted* (*stuA*) were identified and knocked-out by *Agrobacterium*-mediated transformation. Although deletion of the *ZtAbaA, ZtBrlA1*, and *ZtFlbB* genes had no apparent effect on *Z. tritici* asexual sporulation or on pathogenicity, deletion of *ZtFlbC* or *ZtBrlA2* resulted in mutants with reduced pycnidiospore production compared to the parental IPO323 strain. Deletion of *ZtStuA* gave non-pigmented mutants with altered vegetative growth and eliminated asexual sporulation and pathogenicity. These findings suggest that the well-established *A. nidulans* model of asexual sporulation is only partially applicable to *Z. tritici*, and that this pathogen likely uses additional, as yet uncharacterized genes to control asexual sporulation.

## Introduction

The ascomycete fungus *Zymoseptoria tritici* (synonym *Mycosphaerella graminicola*) causes septoria tritici blotch, a major disease of wheat worldwide (Ponomarenko et al., 2011; Dean et al., 2012). There are currently no wheat varieties which are fully resistant to the fungus, so disease is often managed by fungicide application. It has been estimated that 70% of total annual fungicide usage in the European Union is used primarily against *Z. tritici* (Ponomarenko et al., 2011; Torriani et al., 2015), which highlights the importance of this pathogen.

In the field, *Z. tritici* is able to reproduce both sexually and asexually. Asexual reproduction occurs via the formation of asexual fruiting bodies (pycnidia) which produce the asexual spores (pycnidiospores).The pycnidia develop within the substomatal cavity of the infected wheat leaf and mature approximately 21–28 days after initial colonization by the fungus. These fruiting bodies appear macroscopically as dark brown dots against the pale necrotic lesions formed by the fungus (Kema et al., 1996; Dancer et al., 1999; Duncan and Howard, 2000).

Microscopically, pycnidia are typically 60–200 μm in diameter and subglobose in shape, with an ostiole below or protruding through the stomatal pore (Eyal et al., 1987; Kema et al., 1996). Each pycnidium produces an estimated 10,000 pycnidiospores, which are exuded through the ostiole during conditions of high humidity in a gelatinous matrix termed cirrhus (Eyal et al., 1987; Kema et al., 1996; Dancer et al., 1999; Duncan and Howard, 2000; Palmer and Skinner, 2002). The pycnidiospores are then transmitted to the leaves and stems of neighboring host plants by rain splash or direct mechanical contact (Lovell et al., 2004). It is not unusual for almost all substomatal cavities of a lesion to be colonized and contain mature pycnidia. As a result, massive asexual sporulation can occur from a single successful infection, allowing propagation and spread to adjacent plants.

Despite the global importance of this pathogen, to date there has been no detailed investigation into the key genes controlling asexual sporulation in *Z. tritici*. Previous studies focusing on other aspects of the pathogen's biology have occasionally reported defects in sporulation among mutant phenotypes following gene disruption. Examples include the *Z. tritici* Gα subunits *MgGpa1, MgGpa3*, and *MgGpb1* mutants or the *ZtWor1* deletion, all of which have reduced pathogenicity and are therefore unable to form mature pycnidia *in planta* (Mehrabi et al., 2009; Mirzadi Gohari et al., 2014). These results are typically obtained from *in planta* experiments where asexual sporulation has not been separated from virulence, so the impact on sporulation may be a by-product of a reduction in pathogenicity.

The most detailed and in-depth characterization of the genetic basis of asexual sporulation in an ascomycete species has been in the model fungus *Aspergillus nidulans* (reviewed in Adams et al., 1998; Etxebeste et al., 2010; Park and Yu, 2012). The key genes regulating asexual sporulation have been determined through mutant analysis, with clear phenotypes for each step in the process. In *A. nidulans*, asexual spores (termed conidia) are borne on stalk-like multicellular structures called conidiophores. Three central regulatory genes have been identified which together promote successful conidiophore development; *bristle* (which produces two overlapping transcripts, *brlA*α and *brlA*β) (Adams et al., 1988; Prade and Timberlake, 1993), *abacus* (*abaA*) (Sewall et al., 1990; Andrianopoulos and Timberlake, 1994) and *wet white* (*wetA*) (Mirabito et al., 1989; Marshall and Timberlake, 1991).

The *brlA* gene is important for the transition from apical growth of the conidiophore stalk to vesicle formation. Null *brlA* mutants form bristle-like structures with elongated conidiophore stalks, about 20–30 times the normal length, that are unable to form the vesicle or the remaining structures (Clutterbuck, 1969; Adams et al., 1988). The *abaA* gene is activated by *brlA* and is required for phialide differentiation, the phialides being the progenitor cells from which conidia are produced. Mutants in this gene have normal conidiophore stalks but produce abacus-like structures on the vesicle (Clutterbuck, 1969; Sewall et al., 1990). Finally, the *wetA* gene is activated by *abaA* and has been shown to be involved in the proper synthesis of the conidial cell walls. Mutants in this gene produce colorless conidia which autolyse after a few days (Clutterbuck, 1969; Marshall and Timberlake, 1991). Upstream of the central regulatory genes are *fluG, flbA, flbB, flbC, flbD*, and *flbE*. These are all required for proper expression of *brlA* and for the initiation of conidiation. Deletion of these genes results in “fluffy” colonies of profuse aerial hyphae (Wieser et al., 1994). Other genes known to be involved in asexual sporulation in *A. nidulans* are the developmental modifiers *medusa* (*medA*) and *stunted* (*stuA*), which are involved in cell differentiation and patterning of the conidiophore (Clutterbuck, 1969; Martinelli, 1979; Miller et al., 1991, 1992; Adams et al., 1998).

Homologs to the genes regulating asexual sporulation in *A. nidulans* have been identified in the wider Dikarya, suggesting that a conserved set of genes may regulate the patterning of divergent fruiting body structures. For example, *stuA* is evolutionarily widely conserved and homologs with roles in sporulation have been identified in *Ustilago maydis, Parastagonospora nodorum, Fusarium oxysporum, Aspergillus fumigatus, Magnaporthe oryzae, Acremonium chrysogenum*, and *Glomerella cingulata* (Ohara and Tsuge, 2004; Sheppard et al., 2005; Tong et al., 2007; García-pedrajas et al., 2010; IpCho et al., 2010; Nishimura et al., 2014; Hu et al., 2015).

The objective of this study was to assess to what extent the *A. nidulans* model for regulation of conidiation holds true for *Z. tritici*. Potential homologs to the *A. nidulans* genes *abaA, brlA, flbB, flbC*, and *stuA* were selected for gene deletion studies in *Z. tritici*. Gene deletion plasmids were transformed into *Z. tritici* via *Agrobacterium*-mediated transformation, and the resulting knock-out mutants were characterized to assess differences in vegetative growth, pathogenicity, and the ability to sporulate asexually *in vitro* and *in planta*. The findings from this study shed light on some of the key genes required for asexual sporulation in *Z. tritici*.

## Materials and methods

### Comparative genomic analyses in *Z. tritici*

Genes involved in asexual sporulation in the model fungus *A. nidulans* were identified through a literature search using the Web of Sciences^TM^ (http://www.webofknowledge.com) and the National Centre for Biotechnology Information (NCBI) database (http://www.ncbi.nlm.nih.gov). The protein FASTA sequences were BLAST searched against the *Z. tritici* genome database (http://genome.jgi-psf.org/Mycgr3/Mycgr3.home.html) using the tblastn and Filtered Models (transcripts) algorithms.

When more than one BLAST match occurred in *Z. tritici*, the sequence of the original query gene and the *Z. tritici* genes were aligned using Clustal X version 2.0 (Larkin et al., 2007). Molecular Evolutionary Genetics (MEGA) 6 software was then used to create neighbor-joining phylogenetic trees between the original sequence and the observed matches in *Z. tritici*.

### Plasmid construction

Knock-out plasmids were constructed using yeast-based homologous recombination. Primers for plasmid construction are listed in Supplementary Table 1. The plasmids consisted of a pCAMBIA0380YA (yeast-adapted) backbone, two 1.5 kb flanking regions and Hygromycin-*trpC* resistance cassette from pCB1003 (Carroll et al., 1994). The flanking regions and Hygromycin-*trpC* resistance cassette were amplified using Phusion® High-Fidelity DNA Polymerase (Thermo Scientific).

Plasmid DNA was recovered from *Saccharomyces cerevisiae* using the Zymoprep™ Yeast Plasmid Miniprep II kit (Zymo Research) following the manufacturer's instructions. The plasmids were then propagated in *E. coli ccdB* or DH5α cells, and isolated using the Gene JET Plasmid Miniprep Kit (Thermo Scientific) or Gene JET Plasmid Midiprep Kit (Thermo Scientific) following the manufacturer's instructions. Sequencing of plasmids was carried out by GATC Biotech using the LIGHTRUN^TM^ sequencing service with primers listed in Supplementary Table 2.

### *Agrobacterium-*mediated transformation

Knock-out plasmids were transformed into *A. tumefaciens* LBA1126 and AGL1 cells. *Z. tritici* IPO323 and Δ*ku70* strains were transformed by *Agrobacterium*–mediated transformation after Derbyshire et al. (2015).

### Confirmation of knock-out mutants

Initial screening of knock-out mutants were confirmed by growth on YPDA supplemented with Hygromycin B and Timentin. Mutants were sub-cultured at least three times to single colonies, and successful knock-out of the candidate gene confirmed by double PCR. Fungal DNA was extracted using the protocol outlined in (Liu et al., 2000). Double PCR was carried out using two primer pairs; the first primer pair was designed to amplify the wild-type gene, and the second pair to amplify the Hygromycin-*trpC* resistance cassette. Primers used for knock-out confirmation are listed in Supplementary Table 3.

### *In vitro* experiments

Fungal isolates were cultured onto either Czapek Dox-V8 juice (CDV8) agar (46 g/L Czapek Dox agar, 200 ml/L V8® Original vegetable juice (Campbell's), 3 g/L calcium carbonate and 10 g/L technical agar), PDA (24 g/L potato dextrose broth and 20 g/L technical agar), YPDA agar (10 g/L yeast extract, 20 g/L peptone, 20 g/L glucose and 20 g/L technical agar) or wheat extract agar (37.5 g/L homogenized 21 day-old wheat leaves cv. Riband, 20 g/L technical agar). Cultures were incubated under white light or UV-A light (16:8 light:dark cycle) at 20°C for up to 28 days.

*Z. tritici* liquid cultures were grown by inoculating a 250 ml conical flask containing 50 ml PDB with a 10 μl loop of the fungus. The culture was incubated in a shaker at 200 rpm in the dark at 20°C for up to 10 days.

### *In planta* experiments

The attached leaf wheat inoculation procedure used was similar to that described previously by Keon et al. (2007). The susceptible wheat cultivar Riband was used for all experiments. Wheat seedlings were grown in a single line along the edge of a 9” plastic seed tray in Levington® “F2” peat-based compost. The seedlings were grown for about 21 days at an average of 20°C under a 16 h day length.

The first true leaf of wheat plants at growth stages 12–13 (Tottman, 1987) were then held adaxial side up on polystyrene blocks. Wheat leaves were inoculated with spores from seven day-old CDV8 plates suspended in filter-sterilized 0.1% Tween20 at a concentration of 4 × 10 ^6^ spores/ml, using a cotton bud dipped in the suspension (Motteram et al., 2009) The spore suspension was swabbed over the leaf surface ten times. Infected plants were sealed inside a clear 40 μm thick autoclave bag for the initial 72 h of infection at 20°C under a 16 h day length in order to maintain high relative humidity. Following this period of time, the bags were removed and the infected plants remained at conditions of 20°C and a 16 h day length.

Virulence of *Z. tritici* knock-out mutants was assessed by monitoring and recording disease progression every 2–3 days. Disease symptoms on the infected leaves were scored from 1 to 5 using a modified version of the scale outlined in Skinner (2001).

Infected and control leaves were collected at 28 days post infection to compare pycnidia and pycnidiospore production. Leaf sections 4 cm in length were taken starting at the base of the lesion, and subjected to 100% humidity for 48 h to induce release of pycnidiospores. The leaf sections were then immersed in 2 ml SDW and vortexed for 15 s. Ten μl of the suspension was immediately pipetted onto a haemocytometer to count the number of spores released from the pycnidia. This procedure was used for 10 leaf sections per strain of *Z. tritici*.

In order to assess pycnidia number, a 1 cm section was also taken from each leaf, and the area of the leaf was measured using ImageJ software. The number of pycnidia were counted using a dissection microscope, and used to calculate the number of pycnidia per square millimeter (mm^2^).

### Microscopy stains

Light microscopy was used to assess differences in *Z. tritici* vegetative hyphal morphology, pycnidiosospore number and pycnidiospore morphology between the knock-out mutants and parental IPO323 or Δ*ku70* strain. *Z. tritici* vegetative hyphae were obtained from liquid PDB cultures. Pycnidiospores were harvested from the infected leaves collected at 28 dpi.

Specimens were prepared by putting 5 μl of lactophenol cotton blue stain on a glass microscope slide, followed by 5 μl of the fungal cell suspension and a cover slip. The specimen was left to stand for 1 min before microscope analysis.

## Results

### Identification of *Zymoseptoria tritici* sporulation genes using *aspergillus nidulans* homologs

Homologs to the *A. nidulans* genes *abaA, brlA, flbB*, and *stuA* were identified in *Z. tritici* by BLAST search using the *A. nidulans* protein sequences and tblastn searched against the Filtered Models (transcripts) of the *Z. tritici* genome database (http://genome.jgi-psf.org/Mycgr3/Mycgr3.home.html). A single homolog was obtained in *Z. tritici* for *abaA*, however the *brlA, flbB*, and *stuA* genes all gave multiple significant matches (Table 1).

**Table 1:** Summary of the *Aspergillus nidulans* sporulation genes *abaA, brlA, flbB, flbC*, and *stuA*, and all potential homologs identified in *Zymoseptoria tritici*.

***A. nidulans* gene name**	**NCBI protein accession number**	**Protein information**	**Number of BLAST hits in *Z. tritici***	**Protein identification number of BLAST hits in *Z. tritici***	**Alignment score**	**Percentage similarity of *Z. tritici* protein to *A. nidulans* protein (%)**
*abaA* “*abacus*”	XP_658026	TEA/ATTS domain family	1	92404^*^	234	54.9
*brlA “bristle”*	CBF88417	C2H2 Zinc finger	8	100355^*^	169	37.6
				46840^*^	162	54.0
				100278	141	52.1
				89374	122	33.3
				10443	120	40.0
				15680	119	53.8
				31676^*^	108	41.8
				46610	101	30.0
*flbB “fluffy B”*	CBF79600	BZIP-type transcription factor	3	66896^*^	579	52.8
				97280	165	64.6
				35076	110	34.6
*flbC “fluffy C”*	CBF86815	Zinc finger, C2H2 type	7	100355^*^	595	68.6
				14264	353	40.5
				46610	233	35.6
				31676	185	56.4
				100278	139	43.6
				89374	119	45.5
				107126	100	37.3
*stuA “stunted”*	AAA33325	KilA-N domain	2	93828^*^	862	65.1
				48696	159	38.5

Each A. nidulans gene is listed with the protein accession number and protein information collected from the National Centre for Biotechnology Information (NCBI) database (http://www.ncbi.nlm.nih.gov). The A. nidulans protein FASTA sequences were BLAST searched against the Z. tritici genome database (http://genome.jgi-psf.org/Mycgr3/Mycgr3.home.html) using the tblastn and Filtered Models (transcripts) algorithms. The total number of BLAST hits in Z. tritici, protein alignment score and percentage similarity to the original A. nidulans gene are listed. Z. tritici proteins identified for downstream knock-out studies are marked with an asterisk (^*^).

**Figure 1:**
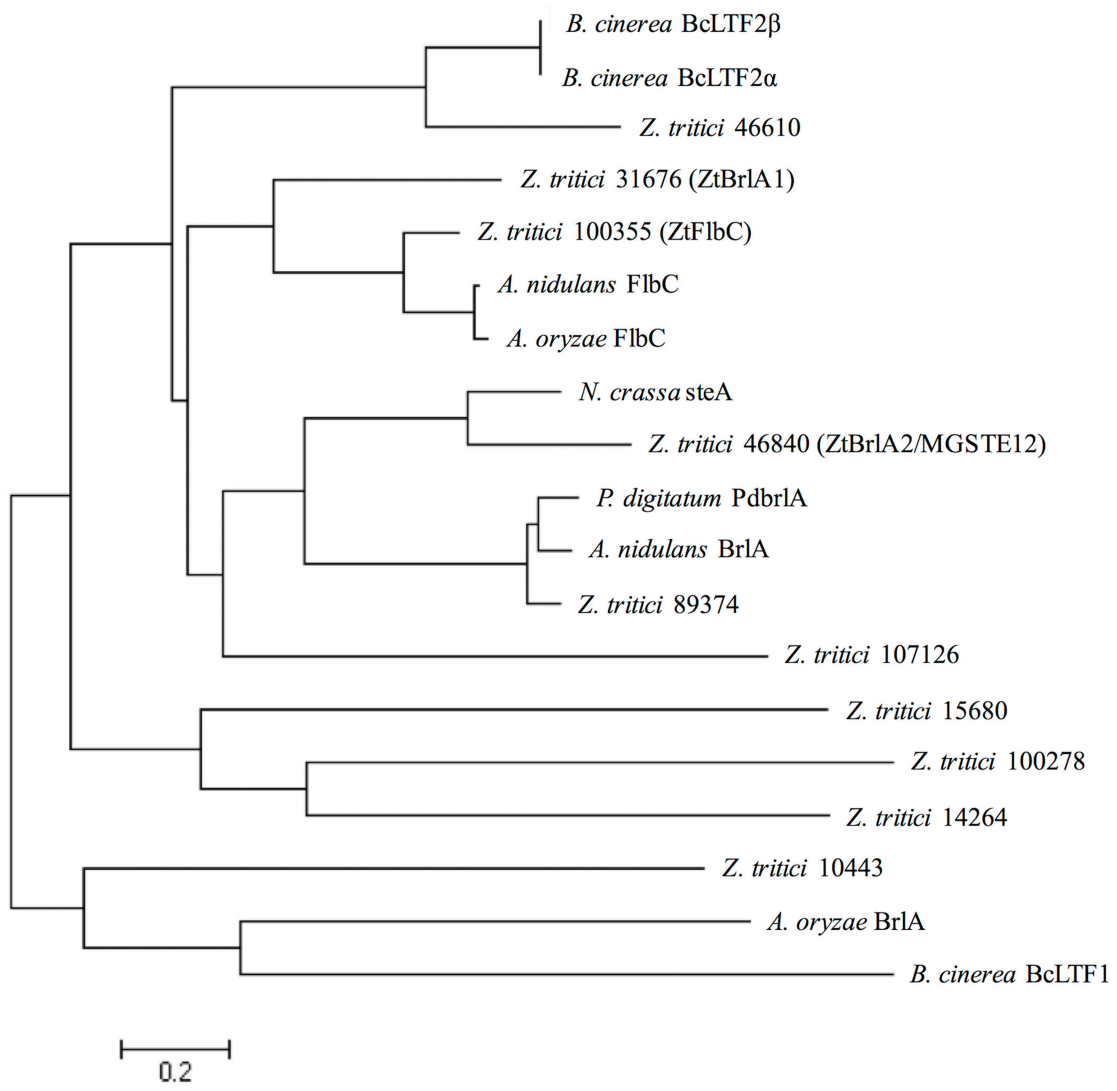
*Zymoseptoria tritici* IPO323 protein sequences in relation to BrlA and FlbC protein sequences from the ascomycete fungi *Aspergillus nidulans, Aspergillus oryzae, Neurospora crassa, Penicillium digitatum*, and *Botrytis cinerea*. Neighbour-joining phylogenetic trees were constructed using FASTA protein sequences obtained from the National Centre for Biotechnology Information (NCBI) database and the *Z. tritici* genome database (http://genome.jgi-psf.org/Mycgr3/Mycgr3.home.html). Branch length represents 0.2 amino acid substitutions per site. The *Z. tritici* protein 100355 shares similarity with FlbC from *A. nidulans*. The *Z. tritici* proteins 31676 and 46840 share similarities with *BrlA*-like proteins from *A. nidulans, P. digitatum* and *N. crassa*.

In order to allow confident identification of the most likely homolog in *Z. tritici*, phylogenetic trees using known sequences for each protein family from *A. nidulans* and the wider Dikarya were used to infer relationships. Where possible, RNAseq data available in the literature were used to validate the *Z. tritici* homologs selected (Yang et al., 2013; Rudd et al., 2015). During the search for *brlA*, the *Z. tritici* protein number 100355 was identified as a match to both *brlA* and the *flbC* proteins (Figure 1). In addition, one of the matches identified, *ZtBrlA2*, was identified as synonymous with the previously identified *MGSTE12* (Kramer et al., 2009). In total, six genes were selected for knock-out in *Z. tritici*, including three potential *brlA* homologs due to the importance of this gene family in controlling asexual sporulation in *A. nidulans* (Table 2).

**Table 2:** The six sporulation genes selected for knock-out in *Zymoseptoria tritici*, listing each gene name, protein ID, transcript size, chromosome location and protein information obtained from the *Z. tritici* genome database.

**Gene name**	**Protein ID Number**	**Transcript Size (bp)**	**Chromosome location**	**Protein Information**
*ZtAbaA*	92404	2,199	chr_4:824620-826874	TEF-1 and TEA transcription factor family
*ZtBrlA1*	31676	294	chr_1:3282702-3282995	Zinc finger, C2H2-type transcription factor
*ZtBrlA2*	46840 (*MGSTE12*)	2,154	chr_8:759342-761612	STE-like transcription factor, Zinc finger, C2H2-type
*ZtFlbB*	66896	1,504	chr_1:4323259-4325858	Basic-leucine zipper (bZIP) transcription factor
*ZtFlbC*	100355	1,614	chr_5:2560859-2562643	Zinc finger, C2H2-type transcription factor
*ZtStuA*	93828	1,515	chr_6:1105154-1106779	APSES domain

*The gene ZtBrlA2 was later identified as synonymous with MGSTE12 (Kramer et al., 2009)*.

### Generation of *Z. tritici* gene deletion mutants

Gene deletion mutants were generated in *Z. tritici* to assess the role of *ZtAbaA, ZtBrlA1, ZtBrlA2, ZtFlbB, ZtFlbC*, and *ZtStuA* in asexual sporulation. Knock-out plasmids were generated in a yeast-adapted version of pCAMBIA_0380 by yeast-based homologous recombination. The knock-out construct consisted of a Hygromycin-*trpC* resistance cassette (indirectly derived from pCB1003; Carroll et al., 1994) flanked by two 1.5 kb regions amplified from either side of the gene of interest, to allow efficient targeting by *Agrobacterium-*mediated transformation (Supplementary Table 1). The Δ*ztabaA*, Δ*ztbrlA1*, Δ*ztbrlA2*, Δ*ztflbB*, and Δ*ztflbC* mutants were successfully generated in the wild-type IPO323 background with 10–40% targeting efficiency. Despite several attempts, we were not able to isolate any Δ*ztstuA* mutants in the wild-type background. However, these were successfully generated in the Δ*ku70* background which had a higher targeting efficiency (Bowler et al., 2010).

Transformants were identified and purified using selective media containing Hygromycin B and confirmed by PCR (Supplementary Figure 1 and Supplementary Table 3). The first primer pair amplified across the flanking region to the IPO323 wild-type gene, and the second pair was used to amplify across the flanking region to the Hygromycin-*trpC* resistance cassette. Successful disruption was indicated by both loss of the wild-type amplicon and gain of the knockout amplicon. Throughout this study, three independent deletion strains and the corresponding parental IPO323 or Δ*ku70* strain were analyzed for each of the genes selected.

### The Δ*ZtbrlA1*, Δ*ZtflbC* and Δ*ZtstuA* mutants have defects in vegetative growth *in vitro*

**Figure 2:**
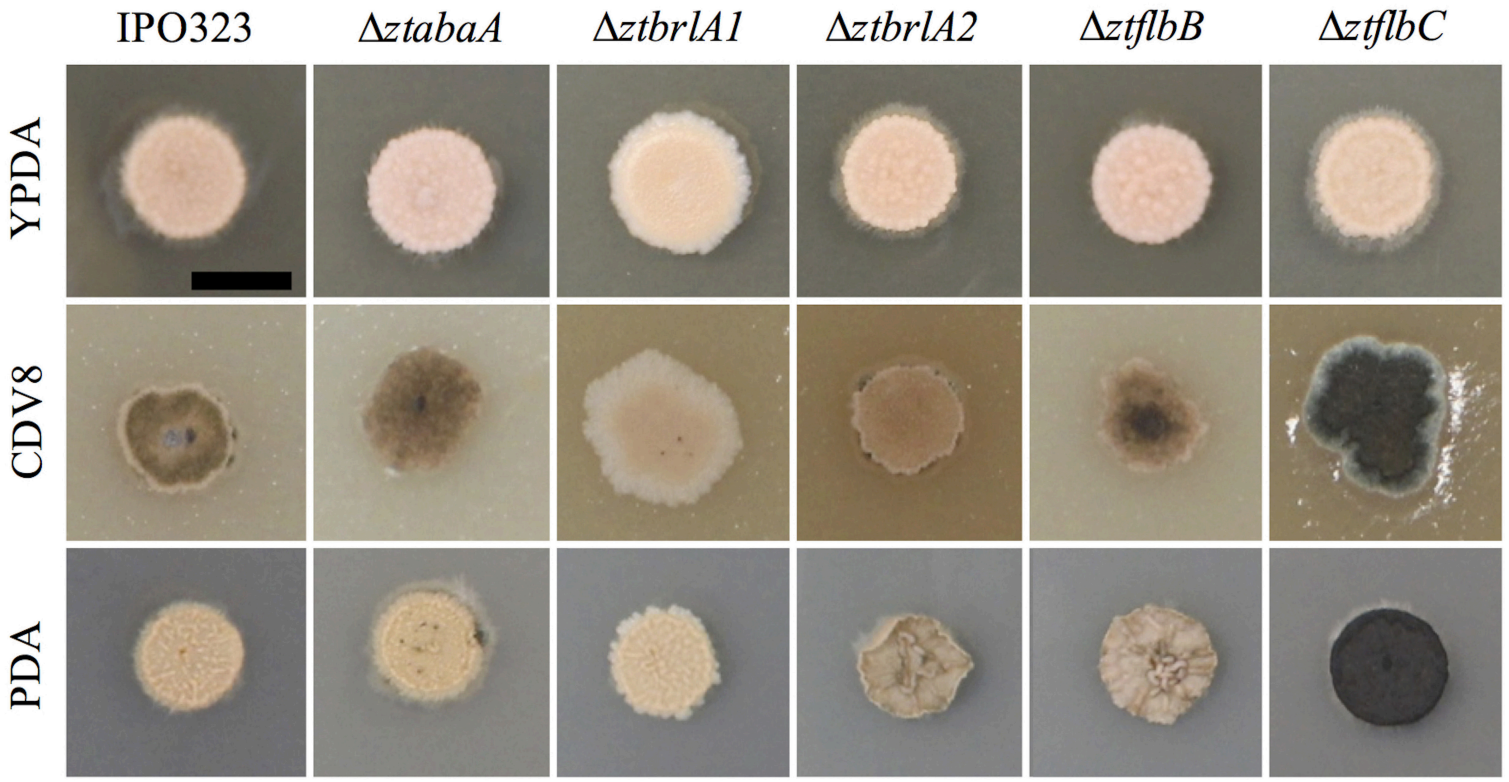
*Zymoseptoria tritici* wild-type (IPO323) and knockout mutants growing on YPDA, CDV8 agar, and PDA at 20°C, under white light (16:8 light:dark cycles), at 7 days post-inoculation (d.p.i.). Images are representative of three independent Δ*ztabaA*, Δzt*brla1*, Δzt*brlA2*, Δ*ztflbB*, and Δ*ztflbc* strains tested for each mutant. Scale bar = 5 mm. The Δ*ztabaA*, Δ*ztbrlA2*, and Δ*ztflbB* mutants are similar phenotypically to IPO323 on all three media types tested. However, the Δ*ztflbC* mutant melanised faster than IPO323 on CDV8 and PDA. The Δ*ztbrlA1* strain melanised slower than the IPO323 strain on all three media types.

Phenotypic differences between the IPO323 or Δ*ku70* parental strain and the deletion mutants were assessed by growing the fungi in liquid PDB and on the solid media PDA, CDV8, and YPDA for 7 days. The Δ*ztabaA*, Δ*ztbrlA2* and Δ*ztflbB* knock-out mutants showed no difference in phenotype to the parental IPO323 strain when grown in liquid PDB or on PDA, CDV8 or YPDA solid media. By 7 days post-inoculation (d.p.i.), all liquid cultures were rosy-pink in color with some melanisation, and microscopy analyses showed all cultures had both yeast-like and filamentous hyphae. On solid media, the Δ*ztabaA*, Δ*ztbrlA2*, and Δ*ztflbB* mutants showed no difference in vegetative growth compared to IPO323 (Figure 2). Although Δ*ztbrlA2* and Δ*ztflbB* vegetative growth sometimes differed to IPO323 on PDA, this was not consistent and was therefore attributed to inherent variation in *Z. tritici* growth.

The Δ*ztflbC* mutant had normal morphology in liquid PDB, however, on solid media PDA and CDV8, colonies of Δ*ztflbC* melanised faster than IPO323 (Figure 2). Unlike the wild-type, the Δ*ztbrlA1* mutant liquid cultures stayed rosy-pink in color and showed no melanisation at seven d.p.i. In addition, the Δ*ztbrlA1* strain also took longer to melanise than IPO323 on all three solid media types tested. Microscopy of liquid culture showed that the Δ*ztbrlA1* vegetative cells were short, unbranching and yeast-like, with only a few starting to undergo the transition to hyphal growth.

**Figure 3:**
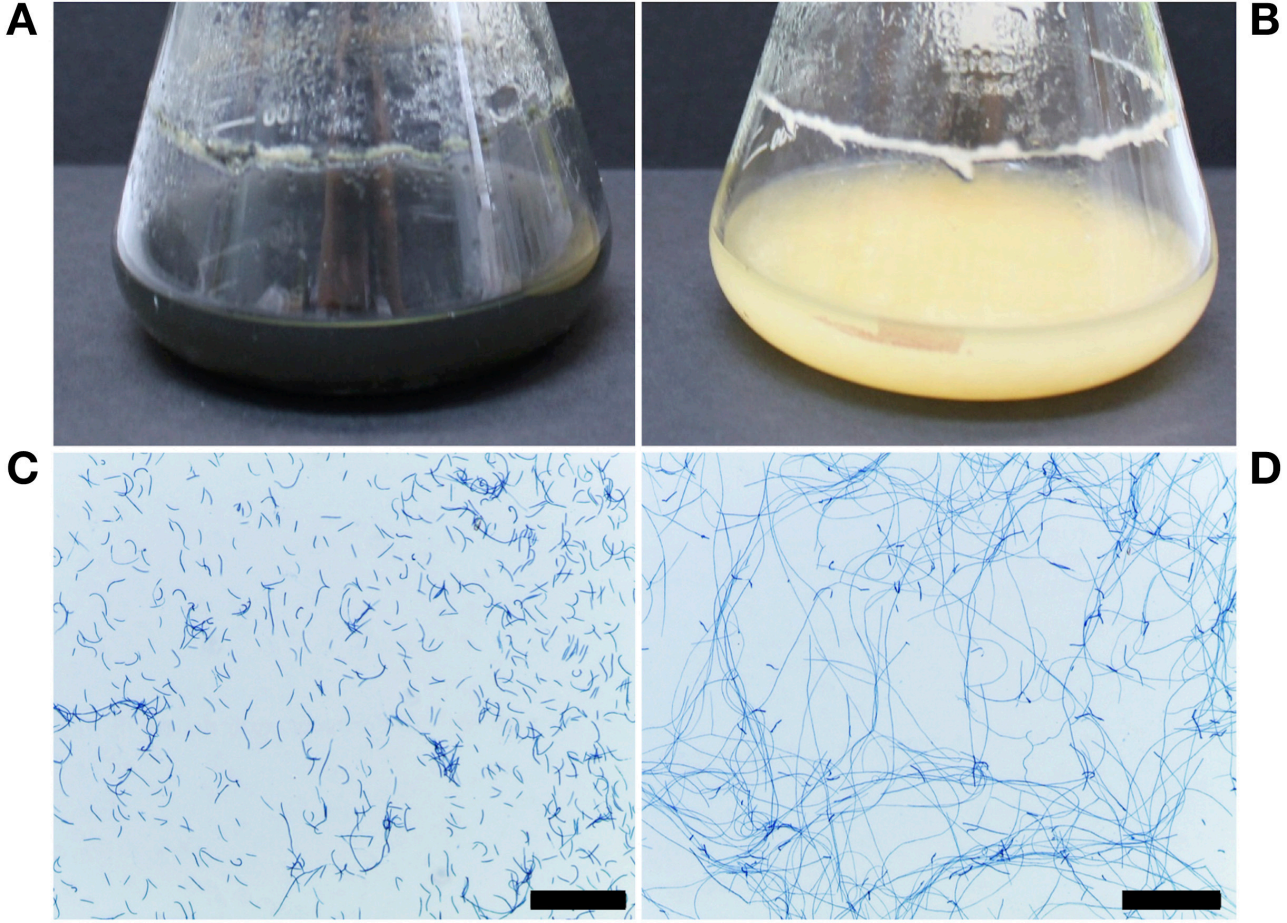
*Z. tritici* Δ*ku70* and Δ*ztstuA* growth in PDB at 20°C on a rotary shaker at 200 rpm, 7 and 10 days post-inoculation (d.p.i.). Images are representative of the three independent Δ*ztstuA* strains tested. Scale bar = 200 μm.; **(A)** Δ*ku70* liquid cultures at 10 d.p.i. are black and melanised **(B)** Δ*ztstuA* liquid cultures at 10 d.p.i. remain pale yellow in color with no melanisation; **(C)** Δ*ku70* cultures at 7 d.p.i. stained with lactophenol cotton blue are yeast-like with some branching hyphal cells; **(D)** Δ*ztstuA* cultures at 7.d.p.i. stained with lactophenol cotton blue have minimal yeast-like growth and instead produce long thread-like hyphae.

The Δ*ztstuA* mutant differed from the Δ*ku70* parent when grown in liquid PDB or on solid media. In PDB, the mutant cultures were pale yellow to rosy-pink in color and did not melanise by 7 d.p.i. The Δ*ztstuA* liquid culture also showed aggregation of the cells to make mycelial pellets. On observation under the microscope, the Δ*ztstuA* mutant cells were not yeast-like, but instead formed a mass of long thread-like hyphae (Figure 3).

**Figure 4:**
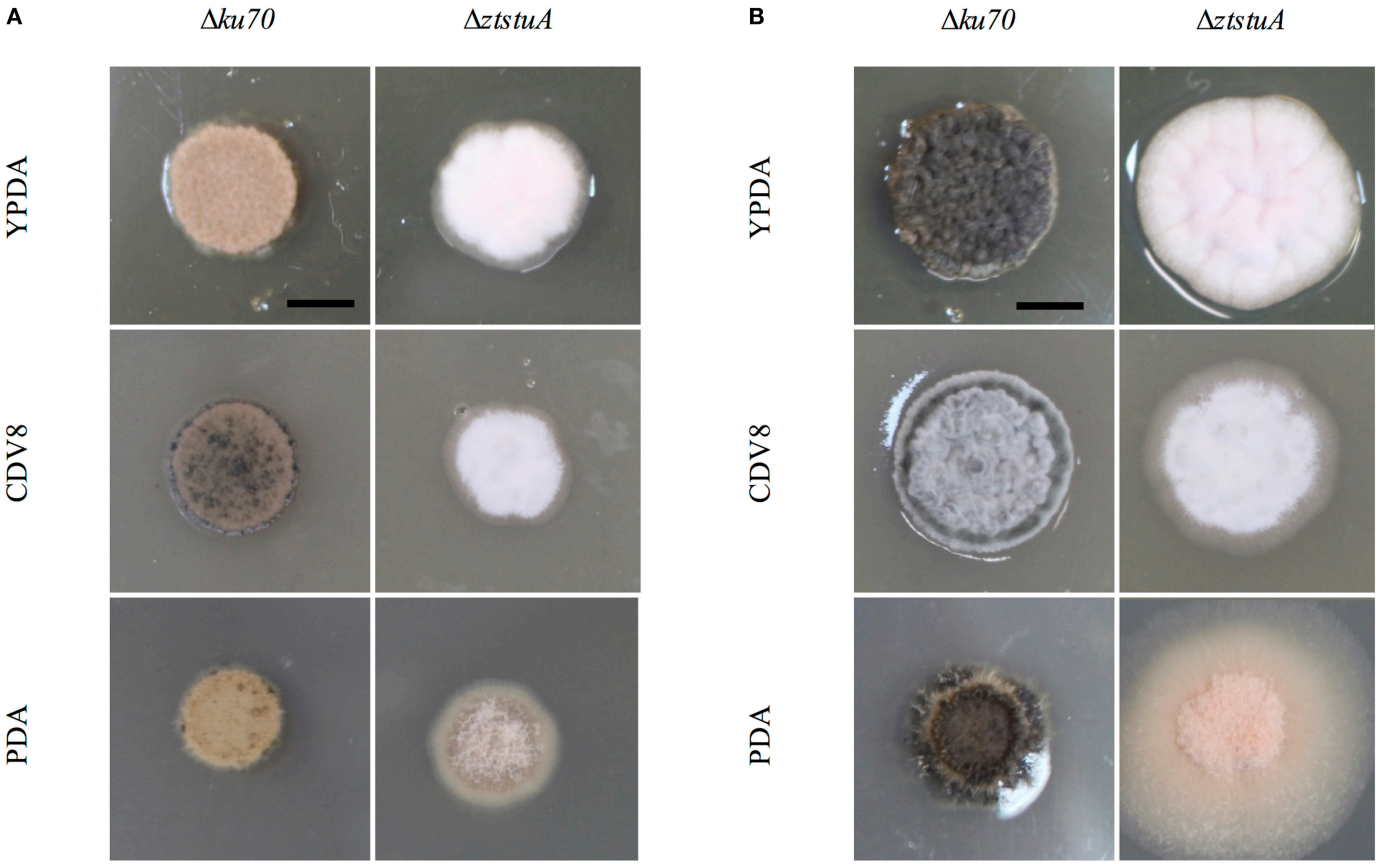
*Zymoseptoria tritici* Δ*ku70* and Δ*ztstuA* growth on YPDA agar, CDV8, and PDA at 20°C, under white light (16:8 light:dark cycles), at 7 days post-inoculation (d.p.i.) and 10 d.p.i. Scale bar = 5 mm; **(A)** at 7 d.p.i. the Δ*ku70* strain grows as rosy-pink yeast-like cells with some melanisation on CDV8 and PDA media. The Δ*ztstuA* mutant grows as a white hyphal colony with no signs of yeast-like growth or melanisation; **(B)** at 10 d.p.i. the Δ*ku70* strain is melanised on all three types of media, but the Δ*ztstuA* strain remains white or rosy-pink in color with aerial hyphae.

When grown on solid media, the Δ*ku70* strain grew as rosy-pink colonies of yeast-like cells which began to melanise by 7 d.p.i. However, the Δ*ztstuA* mutant did not form yeast-like cells and did not melanise, instead colonies of this mutant produced white aerial hyphae on all three types of media tested with no melanisation even after 10 d.p.i (Figure 4).

### Δ*ztstuA* mutants are unable to undergo asexual sporulation *in vitro*

Asexual reproduction was established *in vitro* by incubating the *Z. tritici* mutants and respective background strain on wheat leaf extract agar (WEA) under UV-A light for 28 days. This method was used to test whether the gene deletion mutants could produce pycnidia *in vitro*, as it enables the uncoupling of pathogenicity from asexual sporulation.

By 14 d.p.i. the IPO323 wild-type strain grew lateral hyphae radiating away from the point of inoculation both into and over the agar surface. Leading hyphae at the colony margins formed white hyphal knots by 21 d.p.i. These hyphal knots were 50–200 μm in diameter which formed dense clusters on the agar surface. By 28 d.p.i. the hyphal knots developed into dark brown, globose structures, each with an ostiole. These pycnidial structures were 50–100 μm in diameter and resembled the pycnidia typically observed *in planta*. The structures were present both within and on the surface of the agar, and some oozed a cloudy white liquid which was similar to cirrhus observed *in planta*.

**Figure 5:**
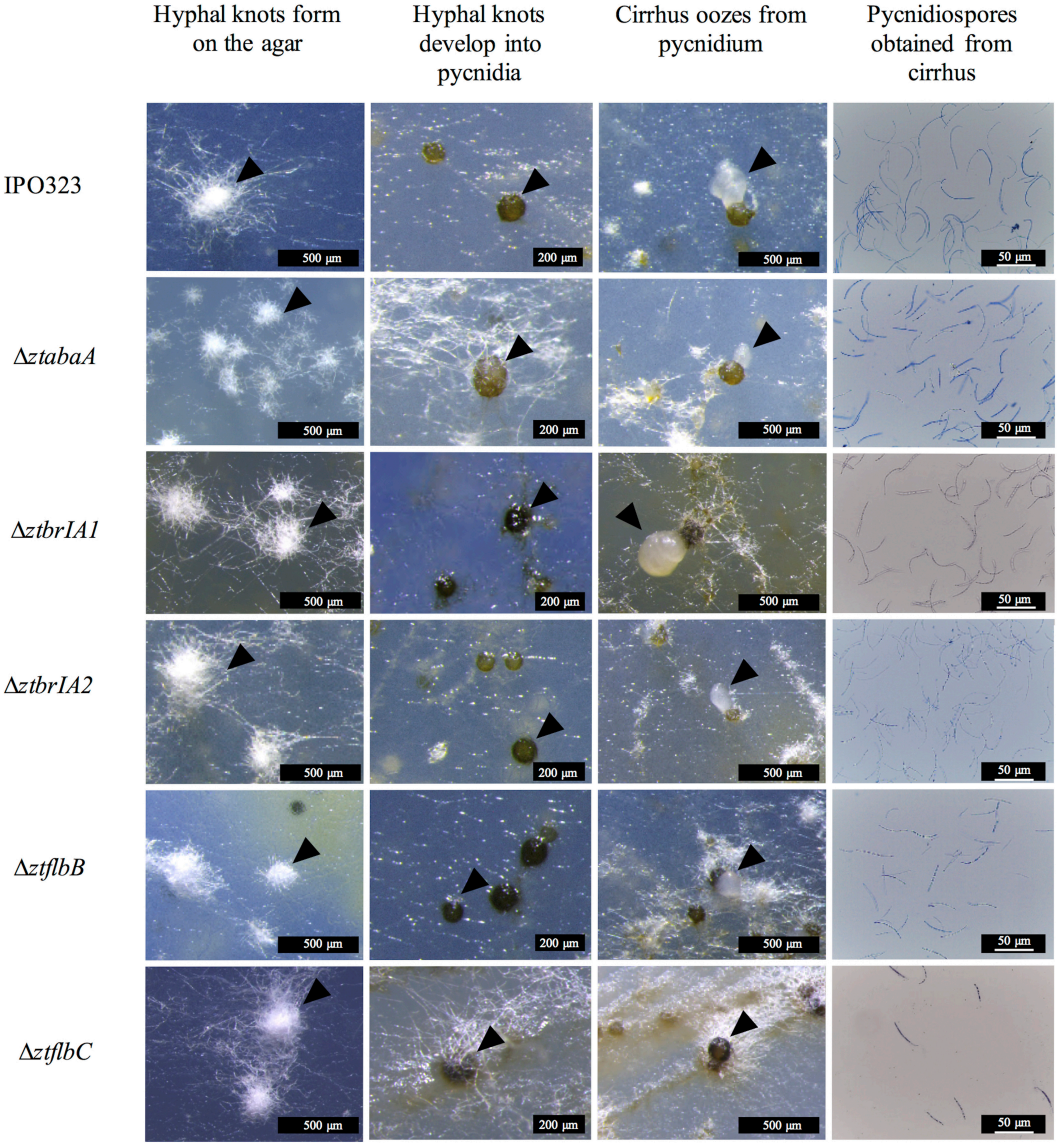
Pycnidia and pycnidiospore production on wheat extract agar (WEA) for IPO323 and selected knock-out mutants, grown at 20°C under UV-A light (16:8 light:dark cycles), 21 – 28 days post-inoculation (d.p.i.). Images are representative of the three independent Δ*ztabaA*, Δ*ztbrlA1*, Δ*ztbrlA2*, Δ*ztflbB*, and Δ*ztflbC* strains tested for each knockout. The different structures formed at each stage of pycnidia development are indicated by black arrows. At 21 d.p.i. IPO323 and Δ*ztabaA*, Δ*ztbrlA1*, Δ*ztbrlA2*, Δ*ztflbB*, and Δ*ztflbC* all develop white hyphal knots on the WEA. The hyphal knots progress to form round brown/black pycnidia by 28 d.p.i. The pycnidia produced by the strains oozed cirrhus, which was extracted and stained with lactophenol cotton blue. The cirrhus obtained from the IPO323 and Δ*ztabaA*, Δ*ztbrlA1*, Δ*ztbrlA2*, Δ*ztflbB*, and Δ*ztflbC* strains all contained curved unbranched pycnidiospores.

The Δ*ztabaA*, Δ*ztbrla1*, Δ*ztbrla2*, Δ*ztflbB* and Δ*ztflbC* mutants were all able to produce pycnidia *in vitro* similar to the IPO323 strain. In addition, the pycnidia produced by the mutant strains followed the same developmental pathway and timings as IPO323 (Figure 5).

The pycnidia-like structures from IPO323, Δ*ztabaA*, Δ*ztbrla1*, Δ*ztbrla2*, Δ*ztflbB*, and Δ*ztflbC* all exuded a cirrhus-like ooze. The cirrhus was collected, stained using lactophenol cotton blue, and observed under a microscope. The cirrhus obtained from the mutant strains contained pycnidiospores which were morphologically indistinguishable from IPO323 pycnidiospores (Figure 5).

**Figure 6:**
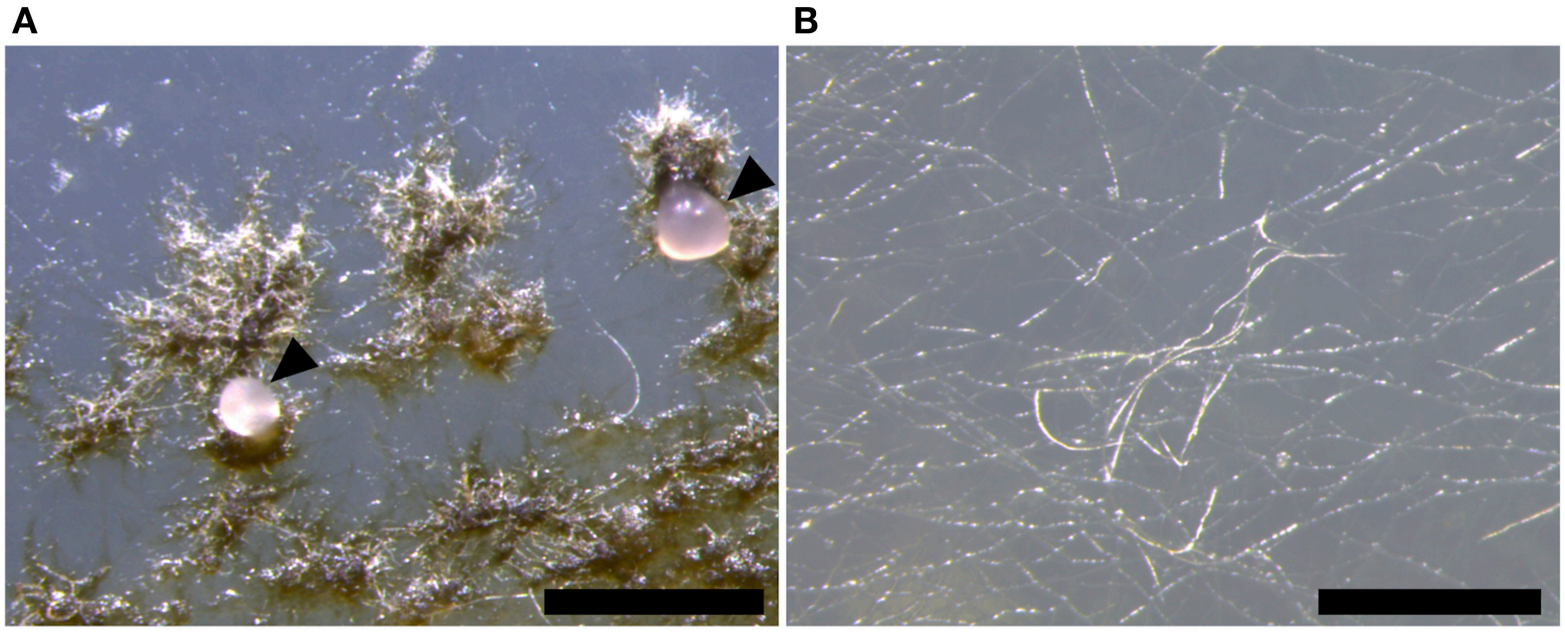
*Zymoseptoria tritici* Δ*ku70* and Δ*ztstuA in vitro* growth on wheat extract agar (WEA) at 20°C under continuous UV-A light at 28 days post-inoculation (d.p.i.). Images are representative of the three independent Δ*ztstuA* strains tested. Scale bar = 500 μm.; **(A)** by 28 d.p.i., the Δ*ku70* strain produces round dark brown pycnidia on the WEA. The pycnidia ooze cirrhus which contains pycnidiospores (indicated by arrows); **(B)** the Δ*ztstuA* strain does not form pycnidia, even after 28 d.p.i. Instead, the Δ*ztstuA* mutant grows as white hyphae across the surface of the WEA.

In contrast, the Δ*ztstuA* mutant did not form hyphal knots, and hence no fruiting bodies or spores under these conditions. Instead, the mutant produced a thin hyphal mat over the surface of the agar, but these did not aggregate into knots or form pycnidia (Figure 6).

### Δ*ZtbrlA2* and Δ*ZtstuA* mutants have reduced virulence *in planta*

In order to assess whether the candidate genes knocked-out in *Z. tritici* have a role in pathogenicity or sporulation, susceptible wheat plants were infected with the mutant strains and IPO323 or Δ*ku70* parental strain. The infection progression was recorded until 28 d.p.i., when the pycnidia were fully developed. Three independent deletion mutants were tested against the respective parental strain for each target gene, and experiments were performed on two independent occasions.

**Figure 7:**
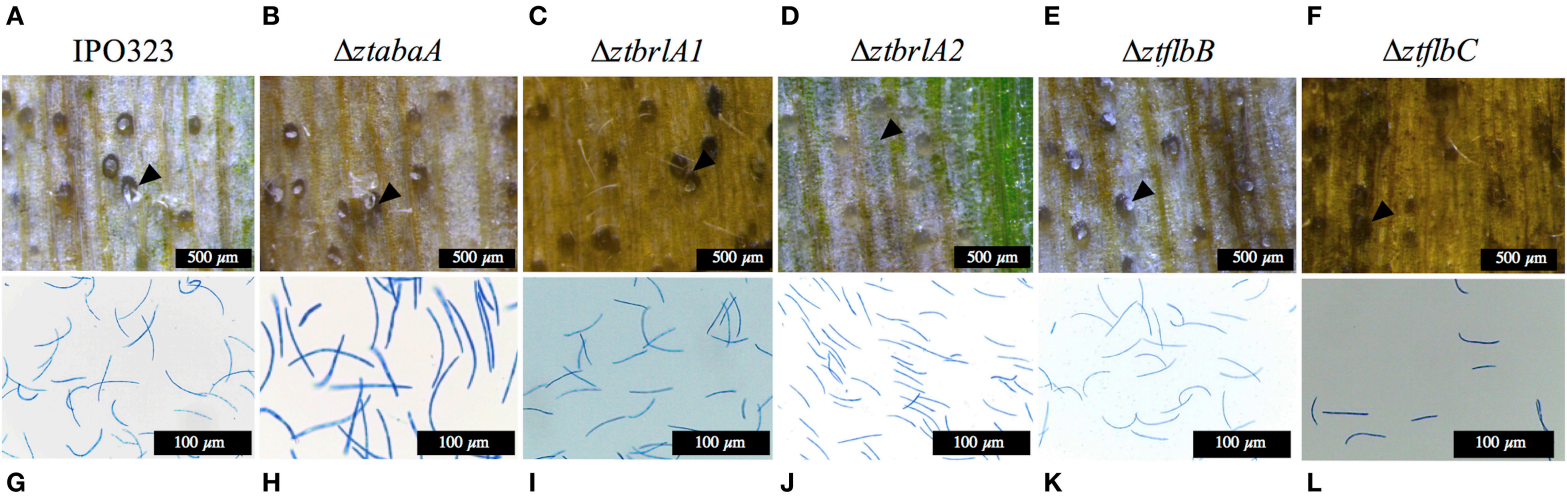
Images of pycnidia (indicated by arrows) and pycnidiospores from wheat leaves infected with 4 × 10^6^ spores/ml from IPO323 and Δ*ztabaA*, Δ*ztbrlA1*, Δ*ztbrlA2*, Δ*ztflbB*, and Δ*ztflbC* mutants. **(A,G)** IPO323; **(B,H)** Δ*ztabaA;* **(C,I)** Δ*ztbrla1;* **(D,J)** Δ*ztbrlA2;* **(E,K)** Δ*ztflbB;* **(F,L)** Δ*ztflbC* at 28 days post-infection (d.p.i.). The knock-out mutant strains were all able to produce pycnidia and pycnidiospores, and these were morphologically similar to the IPO323 strain. The Δ*ztbrlA2* mutant produced fewer pycnidia, and these took longer to develop compared to the IPO323 strain. Fewer spores were harvested from the Δ*ztbrlA2* and Δ*ztflbC* mutants.

Wheat leaves infected with the Δ*ztabaA*, Δ*ztbrlA1*, Δ*ztflbB*, and Δ*ztflbC* mutants showed normal disease progression and normal symptoms. By 28 d.p.i., these lesions contained pycnidia which oozed pycnidiospores under conditions of high humidity (Figure 7). The Δ*ztbrlA2* mutant produced milder symptoms than IPO323, but was still able to make pycnidia and pycnidiospores by 28 d.p.i. To test whether the difference in symptom severity was due to an inability to establish initial infection, the yeast-like cells of the mutant were infiltrated into the leaves using a syringe and disease progression was measured as before. When this method was used, the Δ*ztbrlA2* mutant was able to produce symptoms. The time to chlorosis was the same as for IPO323 delivered by this route, however, the necrotic lesions produced were less severe than those made by IPO323, and these lesions were not as densely covered with pycnidia.

The Δ*ztstuA* mutant was unable to make yeast-like spores on solid culture so the conventional inoculation method was not possible. Therefore, to prepare inoculum, both the Δ*ku70* and Δ*ztstuA* strains were grown in PDB for 7 days. The cultures were then homogenized and the O.D._600_ adjusted to the equivalent of 4 × 10^6^ spores/ml. The cells were harvested by centrifugation and the pellet was resuspended in 0.1% Tween 20. This suspension was used to infect the wheat leaves using a cotton bud.

**Figure 8:**
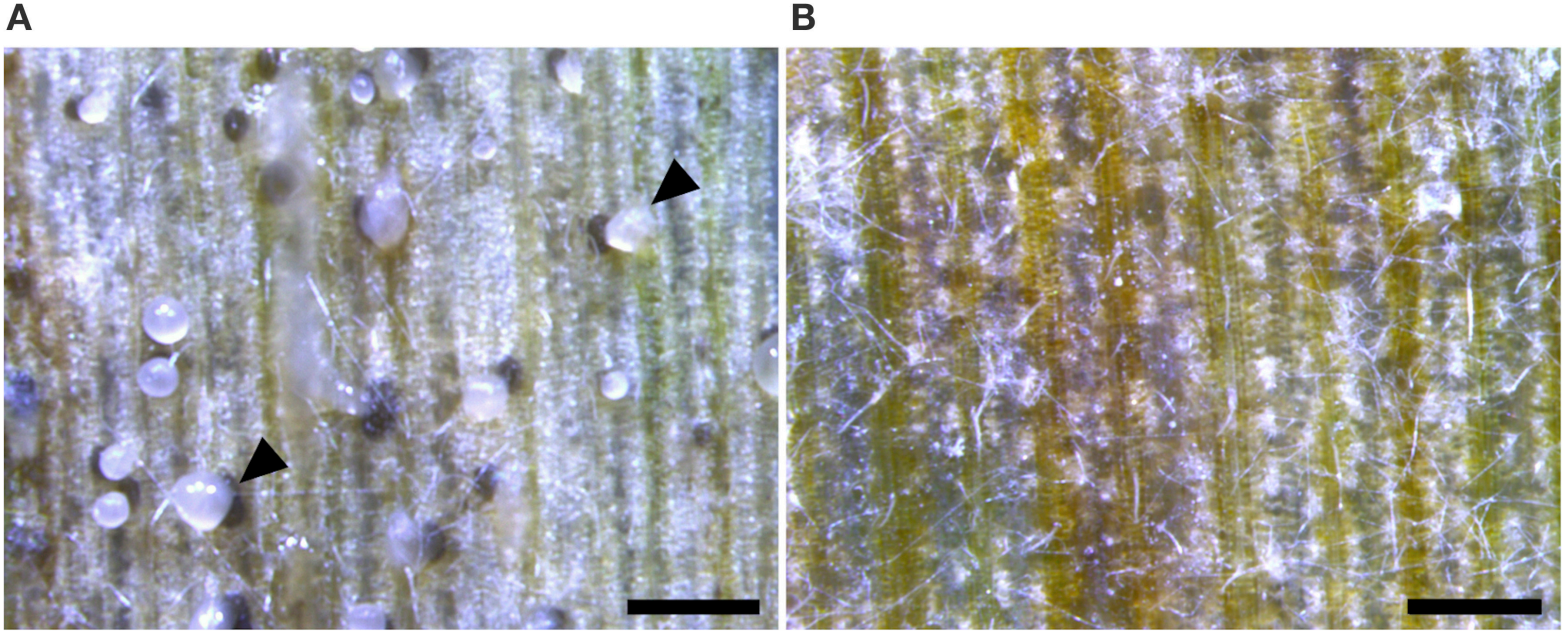
Wheat leaves infected with 4 × 10^6^ spores/ml *Zymoseptoria tritici* Δ*ku70* strain and the Δ*ztstuA* mutant at 28 days post-infection (d.p.i.). Images representative of three independent Δ*ztstuA* strains tested. Scale bar = 500 μm.; **(A)** the Δ*ku70* strain produces necrotic lesions and pycnidia on the wheat leaf. The pycnidia ooze cloudy cirrhus which contains pycnidiospores (indicated by arrows); **(B)** leaves infected with the Δ*ztstuA* mutant show signs of chlorosis but no pycnidia. Instead, the Δ*ztstuA* mutant grows as white hyphae which traverse the leaf surface.

Whilst the Δ*ku70* parent generated symptoms in the normal manner, the Δ*ztstuA* mutant was unable to form productive necrotic lesions on the wheat leaves. Leaves infected with the Δ*ztstuA* mutants did display some patchy chlorosis in the inoculated area and developed occasional small necrotic lesions. Careful inspection of these infected leaves with a dissection microscope showed no evidence of pycnidia within the substomatal cavities of the leaves (Figure 8).

### The Δ*ztbrlA2* and Δ*ZtflbC* mutants have reduced pycnidiospore production *in planta*

**Figure 9:**
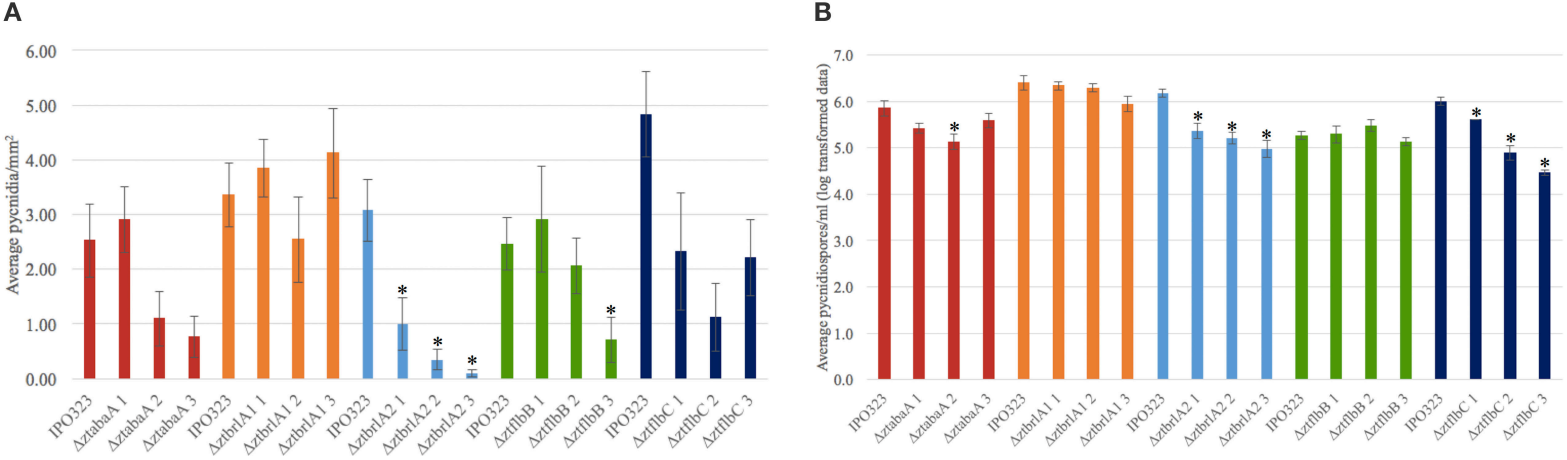
Average numbers of pycnidia and pycnidiospores produced *in planta* by IPO323 and the various mutant strains, assessed at 28 days post-infection (d.p.i.). Three independent knock-out strains were tested for each Δ*ztabaA*, Δ*ztbrlA1*, Δ*ztbrlA2*, Δ*ztflbB*, and Δ*ztflbC* mutant. Bars represent mean SE across 5–10 technical repeats from a single experiment. Bars labeled with an asterisk (*) are knock-out strains with a significant difference in average pycnidia/mm^2^ compared to IPO323 (*p* < 0.05). The Δ*ztbrlA2* strains have significantly reduced numbers of pycnidia/mm^2^ compared to IPO323, however, there is no consistent significant difference between pycnidia/mm^2^ produced by the Δ*ztabaA*, Δ*ztbrlA1*, Δ*ztflbB* or Δ*ztflbC* strains and those made by IPO323. The Δ*ztbrlA2* and Δ*ztflbC* strains have significantly reduced numbers of pycnidiospores/ml compared to IPO323.

Pycnidia numbers and pycnidiospore production of the Δ*ztabaA*, Δ*ztbrla1*, Δ*ztbrla2*, Δ*ztflbB*, and Δ*ztflbC* deletion mutants was compared to the IPO323 strain. There was no significant difference between the numbers of pycnidia or pycnidiospores produced by the Δ*ztabaA*, Δ*ztbrlA1* or Δ*ztflbB* mutant strains. Although Δ*ztflbC* mutants had reduced numbers of pycnidia compared to IPO323, this was not found to be significant (*p* > 0.05). The Δ*ztbrlA2* strain produced significantly fewer pycnidia compared to IPO323 (*p* < 0.05) (Figure 9). In addition, both the Δ*ztbrlA2* and Δ*ztflbC* mutant strains produced significantly fewer pycnidiospores compared to IPO323 (*p* < 0.05) (Figure 9).

Pycnidiospore viability was tested by harvesting the spores from leaves infected with the Δ*ztabaA*, Δ*ztbrlA1*, and Δ*ztflbB* mutants and using them directly to infect fresh plants. These results showed that Δ*ztabaA*, Δ*ztbrlA1*, and Δ*ztflbB* pycnidiospores were able to cause normal disease *in planta* (data not shown). Pycnidiospore viability was not tested for the Δ*ztflbC* orΔ*ztbrlA2* mutants due to low levels of production.

## Discussion

Asexual sporulation by *Z. tritici* enables the pathogen to spread rapidly in the field (Eyal et al., 1987), therefore, targeting production of these pycnidiospores and the pycnidia which make them could be an effective way to reduce disease spread. Although *Z. tritici* makes use of a complex pycnidial fruiting body for asexual spore production, bioinformatic analyses showed that *Z. tritici* has potential homologs to key *A. nidulans* conidiation genes including *abaA, brlA, flbB, flbC*, and *stuA*. The presence of these indicates possible genetic conservation and perhaps the use of a similar pathway to regulate asexual sporulation

Results presented here suggest that the *ZtAbaA* and *ZtFlbB* genes do not have equivalent roles to *abaA* and *flbB* in *A. nidulans*, as deletion did not affect growth or sporulation *in vitro* or *in planta*. We note that RNA-seq data from the literature (Rudd et al., 2015) shows that *ZtAbaA* expression does not change significantly during infection, again perhaps making its involvement in sporulation less likely. *ZtFlbB* expression shows a decrease from 1 to 21 d.p.i. (Rudd et al., 2015), suggesting recruitment of this gene in a different process. Only one homolog to *abaA* was identified in *Z. tritici*, so the only likely candidate was deleted in the fungus. However, two other potential *flbB* homologs exist in the pathogen (Table 1). Therefore, genetic redundancy might explain why deletion of *ZtFlbB* did not affect development or pathogenicity in *Z. tritici*.

The *ZtFlbC, ZtBrlA2*, and *ZtStuA* genes were all shown to have roles in asexual sporulation in *Z. tritici*. In *A. nidulans*, deletion of *flbC* results in delayed and reduced rates of conidiation (Kwon et al., 2010). A similar outcome was seen here in *Z. tritici*, where the Δ*ztflbC* strains had no significant difference in pycnidia production compared to IPO323. However, these mutants did have a significant reduction in pycnidiospore numbers. This could be as a result of a defects in pycnidia viability or ability to release the pycnidiospores. Further evidence for the importance of *ZtFlbC* has been documented in two separate RNA-seq studies, which show a significant change in the expression of this gene during infection, (Yang et al., 2013; Rudd et al., 2015). Therefore, this adds further evidence for the possible role of *ZtFlbC* in a process such as asexual sporulation in *Z. tritici*.

The *brlA* gene is a master regulator of asexual sporulation in *A. nidulans* and is essential for the switch from vegetative growth to asexual sporulation. Null mutants can form the conidiophore stalk, but this grows indeterminately and does not form a mature spore-producing structure. In contrast, over-expression of *brlA* can induce hyphal tips to produce conidiophores (Clutterbuck, 1969; Adams et al., 1988). Whilst the Δ*ztbrlA1* mutant displayed normal asexual sporulation, disruption of *ZtBrlA2* clearly reduced numbers of both pycnidia and pycnidiospores, severely impacting on asexual reproduction.

The milder effects on fruiting body numbers and pycnidiospores seen in the Δ*ztflbC* mutants compared to Δ*ztbrlA2* would agree with findings in *A. nidulans* where *flbC* null-mutants show similar but weaker phenotypes than *brlA* in terms of impacts on conidiation, consistent with the role of FlbC as one of the upstream activators for *brlA* (Kwon et al., 2010).

The deletion of *ZtStuA* shows that this gene is required for asexual sporulation in *Z. tritici*. The Δ*ztstuA* mutants were completely impaired in their ability to make either pycnidia or pycnidiospores, suggesting that *ZtStuA* is essential for this process. The use of the WEA media shows that the lack of sporulation of Δ*ztstuA in planta* is not simply a consequence of the reduced virulence of the fungus, as sporulation was prevented *in vitro* on inductive media. These mutant phenotypes are different to those observed in *A. nidulans*, where *stuA* mutants make abnormal conidiophores and some conidia. However, the results from these studies agree with work carried out on the wheat pathogen *P. nodorum*. The deletion of *SnStuA* from *P. nodorum* results in mutants which fail to produce pycnidia or pycnidiospores. In addition, like Δ*ztstuA* mutants, the *SnStuA* mutants are non-pathogenic and produce thick white aerial hyphae on V8PDA media (IpCho et al., 2010). These similar mutant phenotypes suggest a conserved role of StuA in these two fungi. As *P. nodorum* and *Z. tritici* are both Dothidiomycete cereal pathogens, this could be attributed to their common lifestyle and evolutionary history. Therefore, it could be that both fungal species employ *stuA-*like genes as key regulators of asexual sporulation and pathogenicity, in addition to other un-characterized downstream genes.

Deletion of *stuA* homologs has been carried out in both Ascomycete and Basidiomycete fungi, demonstrating that this gene may have essential roles within the wider Dikarya. For example, deletion of the *Magnaporthe oryzae stuA* homolog, *Mstu1*, results in a reduction in asexual reproduction and attenuated pathogenicity due to defects in appressorium formation (Nishimura et al., 2014). Deletion of the *stuA* homolog, *Ust1*, in *Ustilago maydis* also impacts vegetative growth and pathogenicity (García-pedrajas et al., 2010). Therefore, *stuA* may be a core gene among both the *Ascomycota* and *Basidiomycota* with essential roles in vegetative growth, pathogenicity and sporulation.

**Figure 10:**
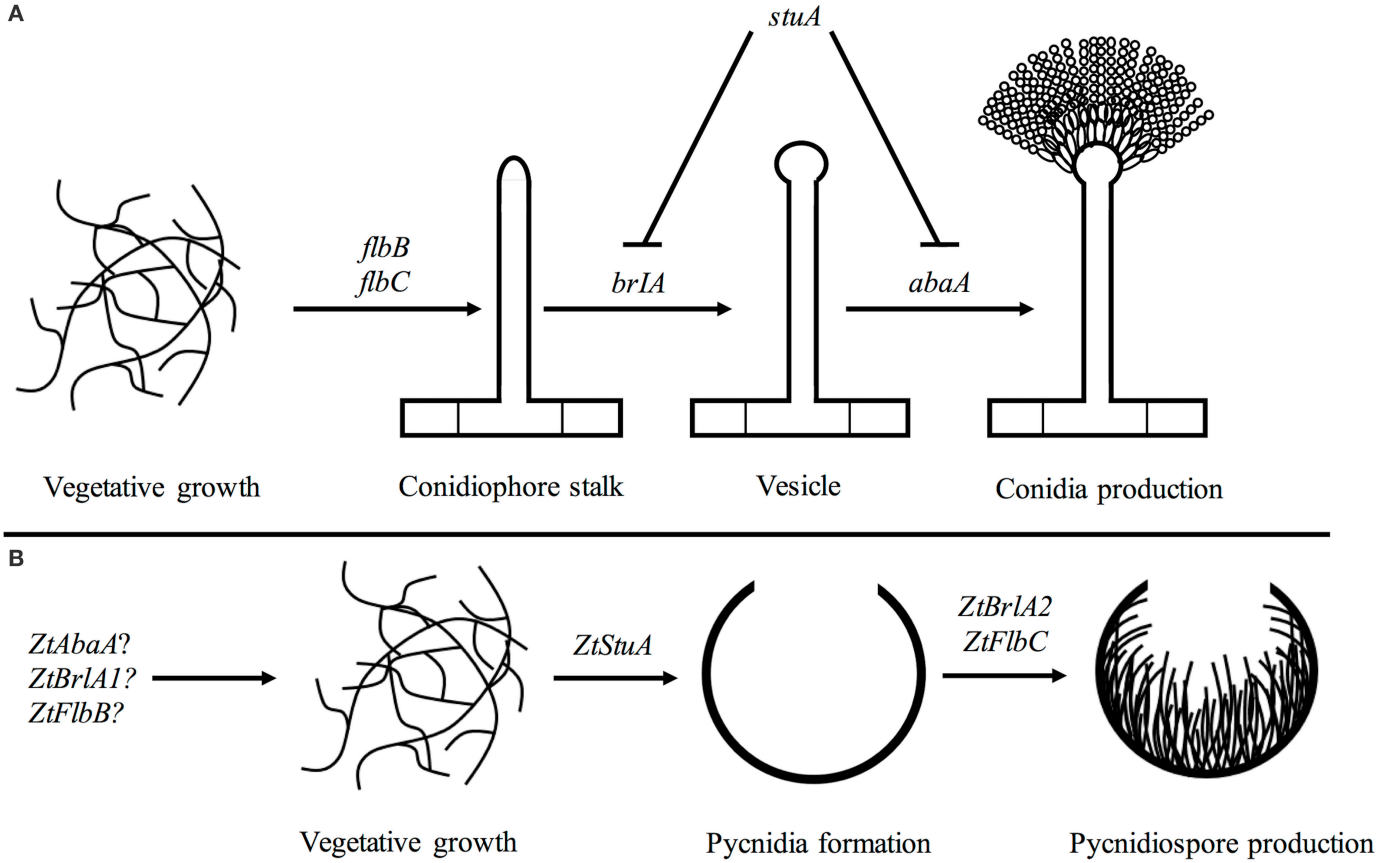
Pathways involved in asexual sporulation in *A. nidulans*, compared to the pathway proposed for *Z. tritici*; **(A)** in *A. nidulans*, asexual sporulation is controlled by the key regulatory genes including *brlA* and *abaA*. These are required for formation of the conidiophore stalk, vesicle and conidia. The *stuA* gene is required for spatial patterning of the conidiophore. The *flbB* and *flbC* genes are required for activation of asexual sporulation (reviewed in Adams et al., 1998); **(B)** in *Z. tritici* the *ZtStuA* gene may encode a key positive regulator of pycnidia production, with *ZtFlbC* and *ZtBrlA2* acting downstream to induce pycnidiospore formation. Disruption of *ZtAbaA, ZtBrlA1*, and *ZtFlbB* had no impact on pathogenicity or asexual sporulation in *Z. tritici*, highlighting differences in regulation of sporulation between *A. nidulans* and *Z. tritici*, however these genes may be involved in other *Z. tritici* processes such as sexual sporulation.

From our initial work here, we propose a rudimentary genetic pathway controlling asexual sporulation in *Z. tritici*. In this pathway, the *ZtStuA* gene encodes an upstream positive regulator of asexual sporulation (ZtStuA). Downstream of *ZtStuA* are the *ZtFlbC* and *ZtBrlA2* genes which encode ZtFlbC and ZtBrlA2 respectively, and these are required for pycnidiospore formation (Figure 10).

It is likely that additional pathways involved in light signaling may also act upstream of *ZtStuA, ZtFlbC* and *ZtBrlA2* and initiate asexual sporulation in *Z. tritici*, however, the interaction of these *Z. tritici* elements is yet to be determined.

Taken together, our findings identify some of the key genes involved in controlling asexual sporulation in this pathogen. We have shown that *Z. tritici* may be using a similar genetic pathway to control initiation of asexual sporulation as *A. nidulans*, however some components of the *Z. tritici* sporulation pathway may differ and could employ currently un-characterized genes. A comparison of the established functions of key *A. nidulans* asexual sporulation genes and the *Z. tritici* homologs examined in this study are summarized in Table 3.

**Table 3:** Comparison of the functions of the key genes regulating asexual sporulation in *Aspergillus nidulans*, and the newly established functions of their homologs in *Zymoseptoria tritici* from the current study.

***A. nidulans* gene**	**Gene function in *A. nidulans***	***Z. tritici* gene**	**Observed gene function in *Z. tritici***
*abacus* “*abaA*”	Initiation of conidiophore phialide cell differentiation (Clutterbuck, 1969; Sewall et al., 1990).	*ZtAbaA*	No role in asexual sporulation, vegetative growth or pathogenicity.
*bristle* “*brlA*”	Initiation of conidiophore vesicle formation. Activation of *abaA* and *wetA* expression (Clutterbuck, 1969; Adams et al., 1988).	*ZtBrlA1*	Transition of vegetative cells from yeast-like growth to hyphal growth. Role in melanisation of vegetative cells. No role in asexual sporulation or pathogenicity.
		*ZtBrlA2* (*MGSTE12*) (Kramer et al., 2009).	Pycnidia and pycnidiospore formation. Required for pathogenicity. No role in vegetative growth.
*fluffy B* “*flbB*”	Initiation of asexual sporulation.Activation of *brlA* expression(Wieser et al., 1994; Etxebeste et al., 2008).	*ZtFlbB*	No role in asexual sporulation, vegetative growth or pathogenicity.
*fluffy C* “*flbC*”	Initiation of asexual sporulation.Conidiospore timing and formation.Hyphal growth and branching.Regulation of *brlA* and *abaA* expression(Wieser et al., 1994; Kwon et al., 2010).	*ZtFlbC*	Pycnidiospore formation. Role in melanisation of vegetative cells under certain *in vitro* conditions. No role in pathogenicity.
*stunted* “*stuA*”	Cell differentiation.Cell wall thickening. Elongation of the conidiophore (Clutterbuck, 1969; Martinelli, 1979; Miller et al., 1991, 1992).	*ZtStuA*	Induction of asexual sporulation. Branching of vegetative cells. Melanisation of vegetative cells. Required for pathogenicity.

*The genes abaA and flbB are essential for proper establishment of asexual sporulation in A. nidulans, but do not have equivalent roles in Z. tritici. The brlA gene is required for conidiophore formation in A. nidulans, but only ZtBrlA2 (MGSTE12) has a role in pycnidia and pycnidiospore formation in Z. tritici. The A. nidulans flbC and stuA genes have roles in asexual sporulation, and the ZtFlbC and ZtStuA genes are also required for this process in Z. tritici*.

The difference in regulatory pathways (e.g., lack of involvement of ZtAbaA or ZtFlbB) used by *Z. tritici* and *A. nidulans* to control asexual sporulation may be explained by the divergent lifestyles and evolutionary histories of the two fungi. *A. nidulans* conidiophores develop as elongated stalks among the colony of exposed hyphae. In contrast, *Z. tritici* pycnidia form within the enclosed space of the plant sub-stomatal cavity. Although the upstream initiator of sporulation, *stuA*, may be shared by these two fungi, downstream regulators may differ because of the variation in fungal fruiting body structure.

From these results we suggest that the *ZtStuA* gene is a key regulator of asexual reproduction in *Z. tritici* which controls initiation of pycnidia development and is likely to be a key regulator in other related species. The *ZtFlbC* and *ZtBrlA2* genes may be downstream regulators which regulate pycnidia and pycnidiospore production. The *Z. tritici* genes such as *ZtStuA, ZtFlbC*, and *ZtBrlA2* could therefore be explored as targets for sporulation inhibitors. The findings from this project therefore help further understanding of an essential developmental process in this under-studied pathogen and open up promising avenues for research in *Z. tritici*.

## Author contributions

AB devised the project, and contributed to experimental design and analysis. AT designed and performed the experiments, analyzed the data, and wrote the manuscript. AB and GF supervised the project and provided feedback on the manuscript.

### Conflict of interest statement

The authors declare that the research was conducted in the absence of any commercial or financial relationships that could be construed as a potential conflict of interest.
