# Supplementary figures and images for: Exploring the Genetic Regulation of Asexual Sporulation in Zymoseptoria tritici
Source: Front Microbiol. 2018 Aug 14;9:1859. doi: 10.3389/fmicb.2018.01859 (PMC6102487; doi:10.3389/fmicb.2018.01859)

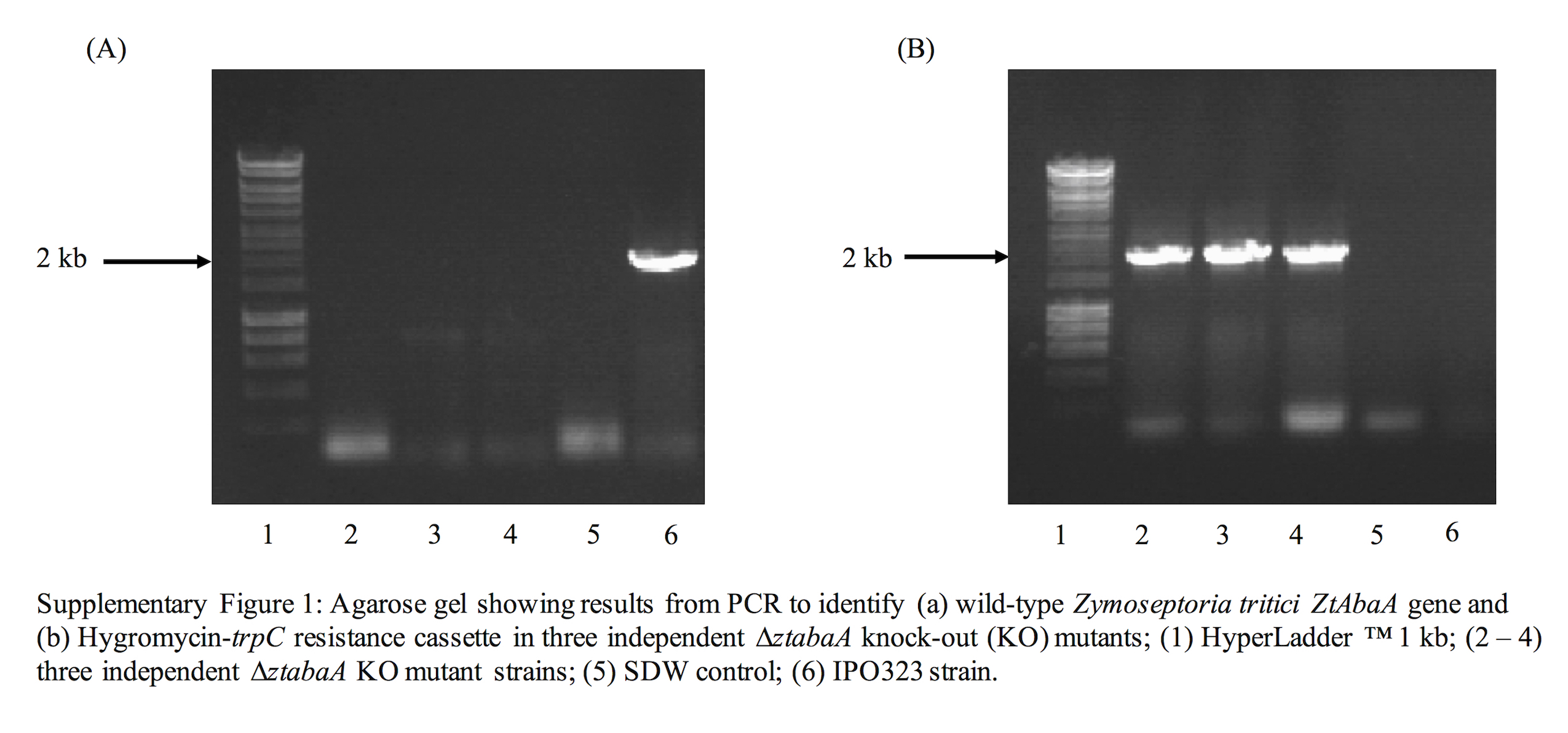

Supplement: Supplementary file 4 [file Image_1.JPEG]
